# Physical activity and subjective well-being of older adults during COVID-19 prevention and control normalization: Mediating role of outdoor exercise environment and regulating role of exercise form

**DOI:** 10.3389/fpsyg.2022.1014967

**Published:** 2022-10-14

**Authors:** Qingqing Yang, Yue Tang, George Jennings, Bin Zhao, Fusheng Zhu, Xiujie Ma

**Affiliations:** ^1^School of Wushu, Chengdu Sports University, Chengdu, China; ^2^School of Foreign Languages, Chengdu Sport University, Chengdu, China; ^3^Cardiff School of Sport and Health Sciences, Cardiff Metropolitan University, Cardiff, United Kingdom; ^4^Chinese Guoshu Academy, Chengdu Sports University, Chengdu, China; ^5^College of Mechanical and Electrical Engineering, Harbin Engineering University, Harbin, China

**Keywords:** physical activity, subjective well-being, older adults, outdoor exercise environment, exercise form, COVID-19 pandemic

## Abstract

Since the outbreak of the COVID-19 pandemic, the physical and mental health of older adults has been threatened. Promoting physical and mental health through physical activity has therefore become a strategy for healthy aging. In order to better understand the impact of the participation of older adults in physical activity, this paper selects different types of physical activity, and examines the relationship between them and subjective well-being through the analysis of the mediation effect of outdoor exercise environment and the regulating effect of exercise form. In this cross-sectional study, a questionnaire survey was conducted in mainland China. The main data come from 903 older adults in five urban areas in Chengdu, Sichuan Province. The surveys were carried out using the Physical Activity Rating Scale, Newfoundland Subjective Well-Being Scale, Exercise Environment Scale, and Exercise Form Scale. SPSS was used for statistical analysis, linear regression analysis was adopted for processing data, and AMOS was used to establish a mediation model. The mediating variable is the outdoor exercise environment, and the moderating variable is exercise form; gender, age, education level, and monthly income were used as control variables. The study results showed that different physical activities (tai chi; health qigong; walking and jogging) were significantly, positively correlated with the subjective well-being of older adults (tai chi: *R* = 0.351, *p* < 0.01; health qigong: *R* = 0.340, *p* < 0.01; walking and jogging: *R* = 0.245, *p* < 0.01); among the activities, tai chi had the strongest effect on the subjective well-being of older adults (*R* = 0.351, *p* < 0.01). Outdoor exercise environment played a mediating role between different physical activity types and subjective well-being of older adults [tai chi: *β* = 0.030, 95% CI (0.005, 0.031); health qigong: *β* = 0.018, 95% CI (0.000, 0.021); walking and jogging: *β* = 0.034, 95% CI (0.008, 0.035)]. Exercise form moderated the subjective well-being of older adults in different physical activities (tai chi: 0.006, *p* < 0.05; health qigong: 0.006, *p* < 0.05; walking and jogging: 0.009, *p* < 0.001). The results of this study demonstrate that the outdoor exercise environment plays a mediating role between different physical activities and the subjective well-being of older adults, and the form of exercise can also moderate the impact of different physical activities on the subjective well-being of older adults. This study has enlightening significance for psychological intervention with older adults facing stress, anxiety and depression.

## Introduction

With the increase in the total number of older adults, population aging has become one of the major problems facing countries around the world (Ferhan and Vesile, 2011). At the end of 2020, China’s population over the age of 60 was 264.01 million, accounting for 18.70% of the national population, of which 190.63 million were aged 65 and over, for an aged rate of 13.50% (National Bureau of Statistics of China, 2021). Obviously, China has become an aged society, and the physical and mental health of older adults has become a topic of universal concern. Since the outbreak of COVID-19, the pandemic has become a major public health emergency (WTTC, 2020), due to the variety of transmission routes, long incubation period, and rapid spread of this highly infectious virus, resulting in tremendous changes in the lifestyle, daily behavior, and life quality of older adults, posing further threat to their physical and mental health. Although the degree of physical weakness and psychological disorders in older adults varies from person to person, relevant studies have found that older adults are more likely than younger people to experience negative emotions during the pandemic (Banerjee, 2020). The psychological disorder detection rate in older adults has increased exponentially, which has made the aged group a main object of psychological counseling and assistance during the pandemic (Xu, 2022). Therefore, a primary task is to reduce adults’ psychological barriers to promote mental health and enhance subjective happiness.

Subjective well-being is an important indicator of mental health and quality of life in older adults (Peterson et al., 2014). It reflects an individual’s overall evaluation of life conditions, including life satisfaction and positive and negative emotions (Diener et al., 2002). Kozma and Stones (1980) conceptualized subjective well-being of older adults as positive affect, negative affect, positive experience, and negative experience. For now, physical activity has been identified as a “meaningful” activity that induces subjective well-being (Tkach and Lyubomirsky, 2006). Physical activity is defined as any bodily movement produced by skeletal muscles that results in energy expenditure (Caspersen, et al.,1985). Studies have shown that regular physical activity is a prerequisite for a happy life in older adults (Sasidharan et al., 2006). First, it can bring positive experiences to older adults and promote happy, optimistic, positive emotions (Heo et al., 2010), improving their subjective evaluation of quality of life satisfaction and overall quality of life (Ragheb and Griffith, 1982); moreover, at the same time, negative states of mind such as depression and sense of worthlessness in older adults can be eliminated (Kim et al., 2017), and the tension brought by negative emotions such as nervousness, anxiety, and anger can be lowered (Lampinen et al., 2006).

At present, it has not been determined what type of physical activities older adults should besty participate in to improve their sense of well-being. Walking and jogging can bring exercise effect to older adults, and are a low-cost and low-impact way to maintain health (Cunningham et al., 2005). It has been proved that walking and jogging can promote the mental health of older adults (Kelly et al., 2017), and can effectively improve their negative emotions (Ekkekakis et al., 2000), thus also improving their quality of life and subjective well-being (Fisher and Li, 2004). Tai chi has the comprehensive characteristics of aerobic exercise, strength exercise, and flexibility exercise (Chang et al., 2010), so it is especially suitable for older adults. Previous studies have found that tai chi can significantly promote the subjective well-being of older adults (Blake and Hawley, 2012), and can reduce their psychological pressure, anxiety, depression and emotional disorders (Wang et al., 2010, 2014). Health qigong is a form of physical and mental exercise (Gallagher, 2003) that cannot only improve the physical function of older adults (Tsang et al., 2002) and enhance the resistance of the human body (Feng et al., 2020) but also help to regulate the mood of older adults (Chow et al., 2012), improve mental health (Rogers et al., 2009; Liu et al., 2010), and make an essential contribution to subjective well-being. Therefore, this paper selects different physical activities (walking and jogging, tai chi, health qigong) that older adults participate in daily to explore their relationships with subjective well-being.

There are many studies on physical activity and subjective well-being during the COVID-19 pandemic, targeting various groups—professional athletes (Schinke et al., 2020), children (Yarımkaya and Esentürk, 2020), middle school students (Okuyama et al., 2021), university students (Wang et al., 2022), and adults (Qi et al., 2020). However, there have been few studies on physical activity and subjective well-being in older adults, focusing mainly on whether physical activity participation has a positive effect on subjective well-being during this specific period (Carriedo et al., 2020; Goethals et al., 2020; Son et al., 2021). Besides, there is a lack of research on how individual subjective well-being is affected and how subjective well-being can be enhanced. Due to the normalization of the pandemic, indoor gyms and other indoor entertainment facilities have become inaccessible (Constandt et al., 2020), which results in limited places for older adults to exercise, so more older adults have chosen to exercise outdoors (Levinger et al., 2022). Studies have demonstrated that they are susceptible to the influences of their surroundings when exercising (Humpel et al., 2002). Outdoor exercise environment is more conducive than indoor ones to improving individual mental health and enhancing subjective well-being (Shanahan et al., 2016). But studies during the COVID-19 pandemic focused primarily on the effects of exercise environments at home on subjective well-being (Mutz and Gerke, 2021), while ignoring the effects of outdoor exercise settings on subjective well-being in older adults. With the adoption of regular pandemic prevention and control, the Chinese COVID-19 slogan of “no gathering together” is deeply rooted in the hearts of the people, and thus the form of physical exercise for older adults in the outdoors will also be affected. Studies have also shown that participation in collective physical exercise in older adults is more helpful than solo exercise for relieving negative emotions and enhancing subjective well-being (Gomez-Cabello et al., 2018). Therefore, the form of exercise should also be one of the main factors affecting the subjective well-being of older adults.

Existing studies fail to explain the subjective well-being of older adults who participate in different physical activities changes due to changing outdoor exercise environment and exercise form during this period, and how its internal mechanisms affect subjective well-being. Based on this, this study hopes to explore the relationship between different physical activities, outdoor exercise environment, exercise form, and subjective well-being during the normalization of the pandemic, as well as their internal mechanisms, so as to provide a reference for the promotion of psychological well-being interventions in older adults.

## Development of hypotheses

### Relationship between physical activity and subjective well-being

Physical exercise, as one of the ways in which older people engage in physical activity, plays an important role in social life (Wang et al., 2018). Relevant studies have confirmed that regular physical activity promotes mental health and helps to improve well-being among older adults (Won et al., 2020) and that the subjective well-being and life satisfaction of older adults who participate in physical exercise are significantly higher than in those who do not participate in physical exercise (Sato et al., 2014; Heo et al., 2016). Moreover, by participating in physical exercise, participants’ subjective well-being can be directly increased, leading to the experience of pleasurable, smooth, peak emotional effects (Peluso and De Andrade, 2005). Studies such as Csikszentmihalyi and Hunter (2014) point out that the longer one participates in physical exercise, the happier one feels.

As age increases, older adults’ body function and athletic capacity decrease, their level of physical activity decreases, and their risk of chronic diseases and fall injuries increases. Therefore, older adults need to perform different types of physical exercise, especially flexibility exercises and step-by-step resistance exercises, to help maintain physical function (Bird et al., 2009; Liu and Latham, 2009). According to related survey results, walking and jogging is the first choice in physical exercise programs for older adults in all regions of China, while tai chi and health qigong are the most popular exercises among older adults (Yang et al., 2019). Walking and jogging can relieve mental tensions, eliminate brain fatigue and tension while walking, and consume calories to control weight (Haskell et al., 2007), making people happy and positive. Subjective well-being can thus be enhanced. Thus, long-term exercise walking and jogging can adjust the human nervous system, stimulate the body and mind, and enhance physical fitness (Kelly et al., 2018). Tai chi and health qigong are traditional elements of Chinese national physical culture, and since their inception, relevant studies have shown that they provide different degrees of improvement on the mental and physical health of exercisers. Previous studies have shown that tai chi can reduce anxiety and depression, improve sleep quality, and elevate spirits while reducing autonomic nervousness and improving neurobehavioral function (Sandlund and Norlander, 2000). Long-term exercise can effectively reduce damage caused by free radicals to the body, improve the metabolic function of cells, delay the aging process to a certain extent, and improve mental health and subjective well-being (Wang et al., 2010). Participation in health qigong helps form positive mood and reduce anxiety and depression (Hui et al., 2006; Johansson et al., 2011). Meanwhile, regular practice of qigong brings mental health, relaxation, improvement in physical ability, and increased subjective well-being (Tsang et al., 2006). Therefore, in this article, the physical activities focused on are walking and jogging, tai chi, health qigong.

Based on this, this study proposes:

*Hypothesis I*: Different physical activities (walking and jogging, tai chi, health qigong) show different level of positive correlations with the subjective well-being of older adults.

### The mediation effects of the outdoor exercise environment

Human beings rely on a favorable environment for survival and development. With the progress of people’s lives, more and more scholars have recognized the importance of environmental influences on physical activity (Kaczynski and Henderson, 2007). The interaction of physical activity with the environment is verified in the theory of socio-ecological systems, which focuses on the relationship between the individual and the environment, emphasizing the significant impact of the environment on human behavior (Sallis and Owen, 1998). Forsyth argue that socio-ecological theory is particularly applicable to older adults, because the environment can influence older adults’ perception and thus their behavior (Forsyth et al., 2009). The exercise environment leaves direct or indirect positive and negative effects on physical activity (Xiong, 2003). The exercise environment is a material prerequisite for older adults to achieve exercise expectations and meet multidimensional needs, and it is also an external cause stimulating enthusiasm and building habits (Van Cauwenberg et al., 2018). The exercise space is a general term for all fitness venues and open spaces suitable for physical exercise, including indoor and outdoor venues and spaces. When older adults participate in physical activity, choice of exercise space will be considered. Existing studies have shown that although exercise venues preferences among older adults are somewhat different across various regions of China, they mainly prefer outdoor places (such as parks, green spaces, squares), supplemented by free indoor venues (Dai, 2017). Other studies have shown that outdoor environments are much safer places to carry out physical activity because the transmission rate of the COVID-19 virus in indoor environments is about 18.7 times higher than that in outdoor environments (Wu et al., 2020). The positive effects of outdoor environment on subjective well-being have been discussed in terms of the comfort, safety, pleasure, and accessibility of the environment (Coon et al., 2011). Participants in outdoor exercise reported relief from tension, confusion, anger, and depression compared to those indoors (Bowler et al., 2010). Therefore, exercising outdoors is more conducive to the subjective well-being of older adults.

Based on this, this study presents.

*Hypothesis II*: Outdoor exercise environment mediate between physical activity and subjective well-being.

### The regulating effects of the exercise form

Physical exercise is accompanied by a certain degree of mental health effect as it produces physiological effects. Given the diversity and complexity of physical exercise form, there are differences in the degree of emotional experience of joy, satisfaction, pleasure, excitement, pride, tension and anxiety that exercisers receive from them, and the psychological effects brought by different exercise forms may also be different. The main ways of physical exercise for the older adults includes group-based exercise and individual exercise. Studies have shown that when older adults participate in collective physical exercise, they expand the scope of their interpersonal interactions and increase their social cognition (Williams and Lord, 1997). Regular participation in collective physical exercise over long periods of time makes it easier to gain social support, improve interpersonal relationships, and gain more mental health (Fox et al., 2007; Orsega-Smith et al., 2007). In the process, they give and receive more material, emotional and spiritual support than those who perform individual exercise. The more collective the form of exercise, the greater the impacts physical activity leaves on the subjective well-being of older adults. That is, collective physical exercise will strengthen the relationship between physical activity and subjective well-being.

On this basis, this study proposes.

*Hypothesis III*: Exercise form moderates the relationship between physical activity and subjective well-being.

**Figure 1:**
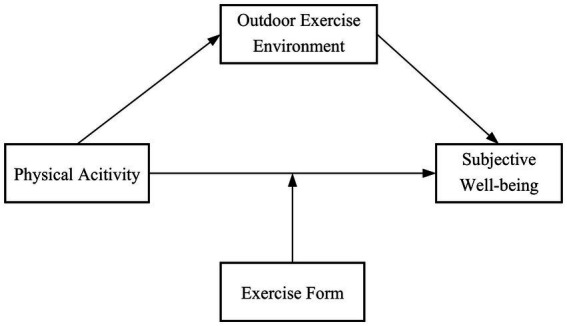
A hypothetical model of physical activity affecting subjective well-being.

In summary, this study hopes to examine the mediation (outdoor exercise environment) and regulation (exercise form) mechanisms working between three different physical activities (walking and jogging, tai chi, health qigong) and subjective well-being (Figure 1), so as to provide a theoretical basis for further explanation of the relationship between different physical activities and subjective well-being and to offer ideas for improving the subjective well-being of older adults.

## Materials and methods

### Study design and participants

From the perspective of economic development, Chengdu ranks in the forefront of Chinese cities and has played an exemplary role in China’s urbanization process. It has been ranked at the top of the list of the happiest cities in China for 13 consecutive years (Dang, 2022). In the process of aging, the proportion of older adults aged 60 and above has come to exceed 17%, and the aging rate has exceeded 13% (National Bureau of Statistics of China, 2021), indicating an aging society. Therefore, Chengdu is selected as the research city, and the results are of great significance to explore the subjective well-being of older adults. From March 15 to May 1, 2022, questionnaires were distributed in five urban areas of Chengdu: Qingyang, Jinniu, Chenghua, Jinjiang and Wuhou. Considering that many older adults find it inconvenient to operate smart phones, in this survey, the questionnaire was mainly on-site, supplemented by an electronic questionnaire published on the “questionnaire star” website; the on-site questionnaire content is truthfully summarized in the database. In order to ensure the rationality of sample distribution, the number of questionnaires in each urban area is fundamentally controlled at about 200.

The selection criteria for this survey include (1) older adults aged 60 and above; (2) older adults with good reading and understanding ability; (3) older adults who have practiced tai chi, health qigong, or walking and jogging; (4) older adults who are willing to participate in the survey after fully understanding the purpose of the survey. Offline exclusion criteria include older adults who have no graphic recognition ability and have hearing impairment; online exclusion criteria include: for questionnaires lacking integrity and authenticity, those with the same answers and a response time of less than 75 s (“questionnaire star” will evaluate the time they spent answering all questions) are regarded as invalid questionnaires and will be removed. Finally, 923 offline questionnaires and 214 online questionnaires were recovered, totaling 1,137. After deleting the questionnaires according to the above exclusion criteria, 903 effective questionnaires were obtained, there were 311 effective questionnaires for walking and jogging, 306 effective questionnaires for tai chi and 286 effective questionnaires for health qigong, with an effective recovery rate of 79.42%. See Table 1 for sample composition.

**Table 1:** Participant demographics.

Demographic category	Frequency	Percent
**Gender**		
Male	447	49.50
Female	456	50.50
**Age**		
60–64	272	30.12
65–69	257	28.46
70–74	202	22.37
75–79	98	10.85
80 and above	74	8.19
**Educational level**		
Primary school and below	406	44.96
middle school	193	21.37
High school or technical secondary school	151	16.72
College (including higher vocational education)	66	7.31
Bachelor degree or above	87	9.63
**Monthly income**		
1,500 and below	305	33.78
1,501–3,500	294	32.56
3,501–5,000	175	19.38
5001and above	129	14.28

### Measurement tool design and reliability testing

#### Physical activity scale

The assessment of the amount of exercise undertaken by older adults uses the physical activity rating scale (PARS-3) revised by Liang (1994). The scale is divided into three dimensions: tai chi, health qigong, and walking and jogging. It examines the amount of exercise in terms of three aspects: intensity, time, and frequency. Amount of Exercise = Intensity × Frequency × Time, in which intensity and frequency are subdivided into 5 grades, recording 1 to 5 points accordingly, and time is subdivided into 5 grades, recording 0 to 4 points. The highest score is thus 100 points, and the lowest score is 0 points. As for the exercise amount evaluation criteria: recording less than 19 is regarded as mild exercise; between 20 and 42, moderate exercise; and after 43 intense exercise. Since tai chi, health qigong and walking and jogging in the Physical Activity Scale are all one-dimensional variables, the structural effect test of the three-factor model composed of nine items using these three variables is examined in this paper, and the fit indices of the model are as follows: χ^2^/24 = 1.2, *p* < 0.001, RMSEA = 0.015, NFI = 0.988, GFI = 0.993, AGFI = 0.987, IFI = 0.988, CFI = 0.998. In addition, the Cronbach’s coefficients of the three sub-scales are 0.755, 0.804, and 0.775, respectively.

#### Subjective well-being scale

Subjective well-being is measured with the Memorial University of Newfoundland Scale of Happiness (MUNSH; 1980). It includes four indicators, namely positive affect (PA), negative affect (NA), positive experience (PE), and negative experience (NE). The scale is scored on 3 points (0 = “no,” 1 = “unclear,” 2 = “yes”). Overall Well-being = PA-NA + PE-NE. It should be noted that this study canceled the reverse scoring setting. That is, the overall well-being score ranges from 0 to 48, and the higher the score, the stronger the subjective well-being. The fit indices for the model are as follows: χ^2^/246 = 1.477, *p <* 0.001, RMEEA = 0.023, NFI = 0.983, GFI = 0.968, AGFI = 0.961, IFI = 0.983, CFI = 0.994, and the Cronbach coefficient is 0.972.

#### Exercise environment scale

With reference to the study of Tan et al. (2020), the exercise environment of older adults was investigated in terms of three aspects: the suitability of the exercise space, the comfort of the exercise atmosphere, and the convenience of the exercise facilities. The suitability of the exercise space includes three questions on issues such as site safety; the comfortable exercise atmosphere element includes three questions on issues such as environmental hygiene; the convenience of exercise facilities section includes two questions, such as the wide distribution of exercise facilities. The question score uses a 5-point Likert scale, which contains five levels from strongly disagree to strongly agree, and is recorded from 1 to 5 accordingly. The Cronbach coefficient is 0.826.

#### Exercise form scale

A question is set: “What kind of physical activities do you do during the long-term control and prevention of Covid-19?” There are 5 options for this question: (1) 100% individual exercise; (2) Ready to try collective physical exercise; (3) If time allows, prefer to participate in collective physical exercise; (4) Considering increasing the frequency of collective physical exercise, and strive to increase that ratio to 100%; (5) 100% collective physical exercise. The 5 options are recoded as 1–5 points respectively, and the higher the score, the higher the tendency to participate in collective activity. Reliability analysis shows that after the introduction of this variable, the Cronbach coefficient reflecting the internal consistency of the questionnaire is 0.907.

#### Control variables

Gender, age, educational background, and monthly income are used as control variables in this study, as these socio-demographic variables have been found to be associated with well-being. A review of relevant studies on well-being reveals that the gender and age of participants have become variables that scholars need to control in their studies (Lehnert et al., 2012; Kim et al., 2021). According to Gerdtham and Johannesson (2001), well-being is significantly associated with factors such as educational background, marital status, and occupation. In addition, Luhmann et al. (2011) found that the relationship between subjective well-being and income is primarily driven by stable differences. The study use gender data as a dummy variable and and the age classifications of Sun (2017), education classification of Zhang (2016), and the China Longitudinal Aging Social Survey (2014) as reference for monthly income options.

### Statistical analysis

After collecting the valid questionnaire data, they were analyzed with SPSS26.0 software. The direct impact of different physical activities and outdoor exercise environment on the subjective well-being of older adults was investigated by correlation analysis and linear regression analysis. The model was validated using the Amos24.0 software package and the structural validity of the scale was verified. At present, the Bootstrap method is a common method for testing mediation effects. The method performs repeated sampling out of the original samples and tests whether the coefficients of the mediation effect are significant, with a 95% confidence interval (Davison and Hinkley, 1997). Therefore, this study used the Bootstrap method to examine whether there is a mediation effect between different physical activities and subjective well-being in outdoor exercise environment. Finally, linear regression was used to test the moderating role of exercise form in the relationship between different physical activities and subjective well-being.

### Validity testing

To further test the convergent validity and reliability of the scale, Average Variance Extracted (AVE) and Construct Reliability (CR) are used as evaluation parameters. From Table 2, AVE of each item is higher than 0.5, proving that convergent degree of the model is acceptable; the CR value of each item is higher than 0.7, proving that the questions in each scale can consistently explain this underlying variable, and all the scales have good construction reliability. In summary, the questionnaires set in this paper present a high degree of reliability and validity.

**Table 2:** Validity and reliability test of the questionnaires.

Variable	AVE	CR
TC	0.507	0.755
HQ	0.578	0.804
W and J	0.535	0.775
OEE	0.544	0.827
SWB	0.695	0.901

CR, composite reliability; AVE, average variance extracted; TC, tai chi; HQ, health qigong; W and J, walking and jogging; OEE, outdoor exercise environment; SWB, subjective well-being.

In order to observe better the effects of different physical activities on subjective well-being, Univariate Analysis of Variance (ANOVA) was used to study the different influences of exercise amount on the items listed on the subjective well-being Scale, that is, PA, PE, NA, and NE. From Table 3, it can be seen that different exercise amount samples influence the four dimensions of the subjective well-being scale significantly (*p* < 0.05), showing that different exercise amount samples had varying differences on PA, PE, NA, and NE. Figure 0.01 (*F* = 61.801, *p* < 0 0.001) shows that the amount of exercise influences subjective well-being significantly. By comparing means between subjective happiness and its four dimensions, the result is moderate exercise > intense exercise > mild exercise.

**Table 3:** ANOVA results of the effects of physical exercise on subjective well-being in older adults.

Variable	Exercise amount (M ± S.D.)			*F*	*p*
	Mild exercise	Moderate exercise	Intense exercise		
SWB	15.66 ± 2.02	33.96 ± 2.18	27.24 ± 2.16	61.801	0.000**
PA	3.72 ± 0.58	8.28 ± 0.65	6.84 ± 0.62	40.851	0.000**
PE	4.38 ± 0.60	8.28 ± 0.66	6.78 ± 0.63	33.137	0.000**
NA	3.48 ± 0.59	8.70 ± 0.61	6.66 ± 0.62	64.23	0.000**
NE	4.14 ± 0.61	8.70 ± 0.62	6.90 ± 0.60	50.677	0.000**

PA, positive emotion; NA, negative emotion; PE, positive experience; NE, negative experience; M, Mean, S.D., Standard Deviation.

** *p* < 0.01.

## Results

### Descriptive statistics for study variables

The means, standard deviations, and correlation coefficients for each of the main variables are shown in Table 4. Significant positive correlations of different physical activities with subjective well-being can be seen (TC: *R* = 0.351, *p* < 0.01; HQ: *R* = 0.340, *p* < 0.01; W and J: *R* = 0.245, *p* < 0.01). In addition, Different physical activities showed positive correlations with outdoor exercise environment (TC: *R* = 0.128, *p* < 0.01; HQ: *R* = 0.079, *p* < 0.05; W and J: *R* = 0.158, *p* < 0.01), and outdoor exercise environment and subjective well-being also showed a positive correlation (*R* = 0.297, *p* < 0.01). In addition, Table 4 also shows that forms of exercise are strongly correlated with different forms of physical activity and subjective well-being, with correlation coefficients being 0.455 and 0.044, and significance levels all below 0.01. Overall, the three hypothesis, H1, H2, and H3, are initially supported.

**Table 4:** Descriptive statistics and correlations for primary variables.

Variable	M	S.D.	TC	HQ	W and J	OEE	EF	SWB
TC	26.422	24.156	1					
HQ	26.323	25.023	0.001	1				
W and J	26.12	24.183	−0.03	0.032	1			
OEE	3.288	0.862	0.128**	0.079*	0.158**	1		
EF	3.419	1.51	0.06	0.049	0.055	0.455**	1	
SWB	5.102	2.309	0.351**	0.340**	0.245**	0.297**	0.044	1

*n* = 903; M, mean; S.D., standard deviation; TC, tai chi; HQ, health qigong; W and J, walking and jogging; OEE, outdoor exercise environment; EF, exercise form; SWB, subjective well-being.

* *p* < 0.05;

** *p* < 0.01.

### Analysis of model mediation effects

**Figure 2:**
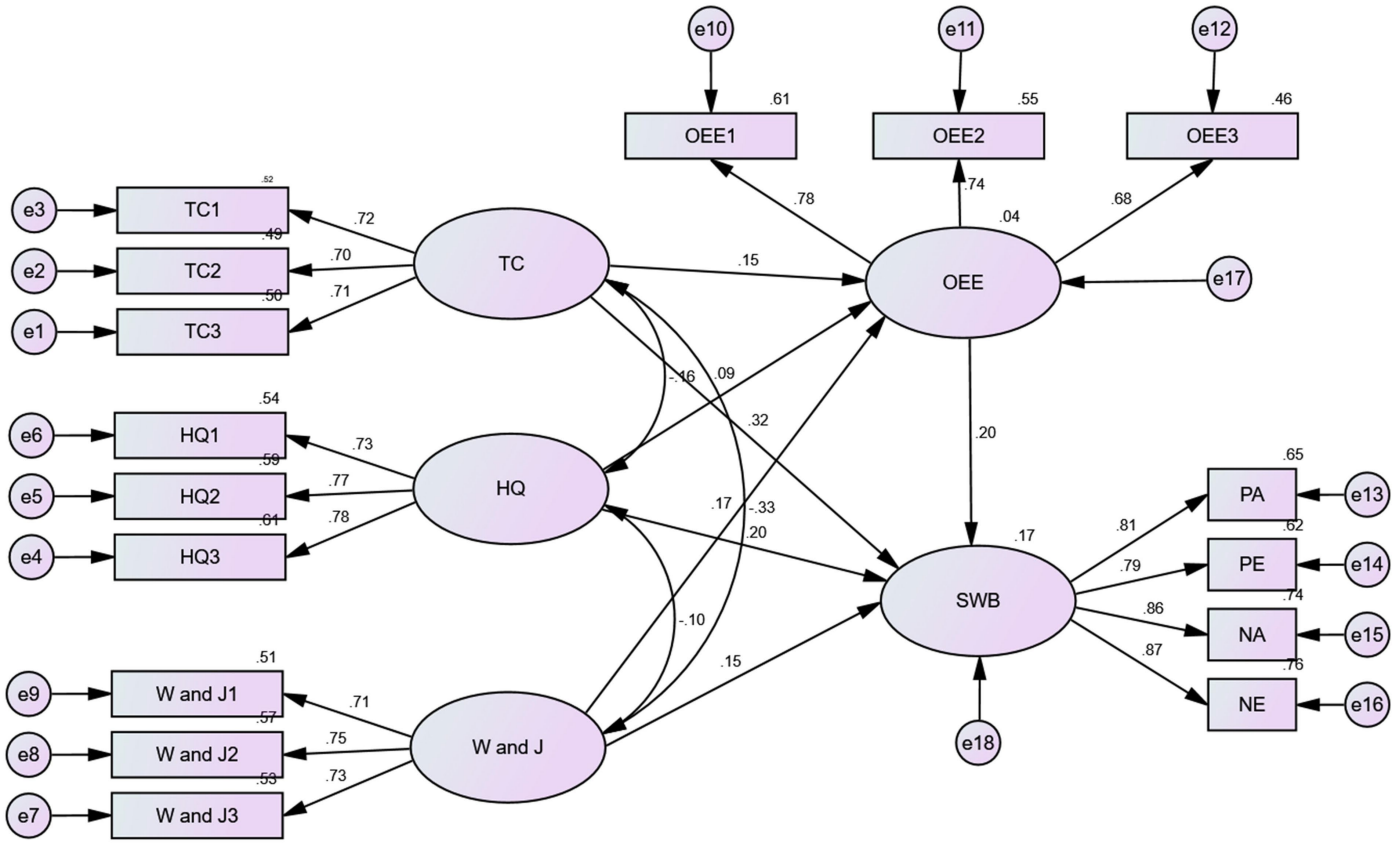
The structural equation model regarding the mediation effects of outdoor exercise environment on the association between different physical activity and subjective well-being. OEE, outdoor exercise environment; SWB, subjective well-being; TC 1-TC 3, Three parcels of evaluative concerns on the intensity of Tai Chi exercise; HQ 1-HQ 3, three parcels of evaluative concerns on the intensity of Health Qigong exercise; W and J 1-W and J 3 three parcels of evaluative concerns on the intensity of walking and jogging exercise. All the path coefficients are standardized.

In order to verify the mediation role of outdoor exercise environment between different physical activities and subjective well-being, the fit analysis of the conceptual framework mediation model is analyzed by AMOS24.0 software package; the standardized path coefficient model of subjective well-being as affected by different physical activity and outdoor exercise environment is shown in Figure 2. According to Hou and Chen (1999), the fit indices of the model generally takes the Chi-Squared (χ^2^) test value *p* > 0.05, χ^2^/df < 3, GFI, NFI, CFI, IFI, and AGFI >0.9, RMSEA <0.05 as reference standards. The results of the fit analysis of the moderation effect model for different physical activities, outdoor exercise environment, and subjective well-being are shown in Table 5, and the various parameters of model fit present an excellent level, indicating that the mediation model in which different physical activities improve the happiness of older adults is reasonable. In Figure 2, the path coefficients (TC: *β* = 0.030; HQ: *β* = 0.018; W and J: *β* = 0.034) of different forms of physical activity → subjective well-being are significant, indicating that different physical activity formats has a direct effect on subjective well-being, proving that H1 is true. The path coefficients of different physical activities → outdoor exercise environment (TC: *β* = 0.15; HQ: *β* = 0.09; W and J; *β* = 0.17) and of outdoor exercise environment → subjective well-being (*β* = 0.20) were both significant, indicating that different physical activities have a mediation effect between the outdoor exercise environment and subjective well-being, proving that H2 is true.

**Table 5:** Model fit indices of mediation effect of different physical activity, outdoor exercise environment, and subjective well-being.

	χ^2^	d*f*	χ^2^/d*f*	*p*	GFI	AGFI	CFI	NFI	IFI	RMSEA
Model	115.094	109	1.056	0.326	0.985	0.979	0.999	0.981	0.999	0.008

Hayes (2009) proposes that in the Bootstrap mediation effects test, at least 1,000 repeated samples out of the original samples are needed. If Bootstrap mediation effects test results show that the Bootstrap test CI does not contain a value of 0, it means that the indirect effect is proven (Zhao et al., 2010). In this study, the mediation effects test was performed by estimating the Booststrap 95% CI for mediation effect by sampling 2000 samples; the results are shown in Table 6. Point estimates that the indirect effects of the different physical activities → outdoor exercise environment → subjective well-being were 0.017, 0.010 and 0.021 respectively, and standard deviation (S.D.) 0.007, 0.005, and 0.007 respectively; Z-values were 2.429, 2.000 and 3.000, respectively. The indirect effects of the Bootstrap 95% CI produced by this path did not contain the value 0, indicating that the mediation effect between different physical activities and subjective well-being in outdoor exercise environment was significant. The point estimates of the direct effects of different physical activities → subjective well-being were 0.206, 0.112, and 0.089, the standard deviations were 0.030, 0.022, and 0.028, and the Z-values were 6.867, 5.091, and 3.179, respectively. The direct effects of the Bootstrap 95% CI produced by this path did not contain 0. The point estimates of the total effects of different physical activity → subjective well-being were 0.223, 0.123, 0.110; the standard deviation was 0.030, 0.023, 0.028; and Z values were 7.433, 5.348, 3.929 respectively, and the total effects of Bootstrap 95% CI produced by that path did not contain 0. This shows that the direct and total effects of outdoor exercise environment → different physical activities are significant. In addition, comparing the total effect sizes of tai chi, health qigong, walking and jogging on subjective well-being, it can be found that different physical activities in terms of their respective influence on the subjective well-being of older adults are ranked as follows: tai chi > health qigong > walking and jogging.

**Table 6:** Test results of mediation effects.

	Path	Point estimate	S.D.	Z	Bootstrapping				Effect size
					Bias-corrected		Percentile		
					Lower	Upper	Lower	Upper	
**Indirect effect**	TC → OEE → SWB	0.017	0.007	2.429	0.005	0.031	0.006	0.033	0.030
	HQ → OEE → SWB	0.010	0.005	2.000	0.000	0.021	0.001	0.022	0.018
	W and J → OEE → SWB	0.021	0.007	3.000	0.008	0.035	0.010	0.038	0.034
**Direct effect**	TC → SWB	0.206	0.030	6.867	0.148	0.266	0.147	0.266	0.320
	HQ → SWB	0.112	0.022	5.091	0.072	0.158	0.070	0.157	0.200
	W and J → SWB	0.089	0.028	3.179	0.036	0.148	0.036	0.148	0.150
**Total effect**	TC → SWB	0.223	0.030	7.433	0.164	0.284	0.164	0.284	0.350
	HQ → SWB	0.123	0.023	5.348	0.080	0.171	0.080	0.170	0.218
	W and J → SWB	0.110	0.028	3.929	0.056	0.169	0.055	0.166	0.184

The Bootstrap sample size is set at 2000. TC, tai chi; HQ, health qigong; W and J, walking and jogging; OEE, outdoor exercise environment; SWB, subjective well-being.

### Analysis of model regulation effect

H3 in this paper assumes that the more collective the form of exercise is, the greater the influence of different physical activities on subjective well-being. We used the three-step test method of Hierarchical Moderated Regression (HMR) analysis and the interaction terms of variables to test the regulation effects. Specifically, we used the following steps for empirical testing, using SPSS26.0 to statistically analyze different physical activities (tai chi, health qigong, walking and jogging) separately. On the basis of testing for common method variance, a correlated analysis is used for initial hypothesis testing, followed by linear regression analysis for regulation model test. It is divided into three linear regression models. First, in model 1 (M1), gender, age, education level, and monthly income are used as control variables, and different physical activity scores are used as independent variables for regression fit; next, model 2 (M2) adds a regulatory variable to model 1 (M1); finally, M3 adds a product term of the independent variable and the adjustment variable to M2. According to the recommendations of Frazier et al. (2004), all predictors are centralized in each model, and the variance inflation factor of all predictors is not higher than 1.17, so there is no multicollinearity problem.

For Model 1, the aim was to investigate the influence of the independent variable (different physical activities) on the dependent variable (subjective well-being) without considering the interference of the regulatory variable (exercise form). From Table 7, it can be seen that different physical activities show significance (TC: *t* = 7.174, *p* < 0.001; HQ: *t* = 6.492, *p* < 0.001; W and J: *t* = 2.041, *p* < 0.05). This implies that different physical activity has a significant impact on subjective well-being, further supporting the H1. Based on an examination of the interaction of different physical activity and exercise forms on subjective well-being (M2 and M3), the results showed that *F* value changed significantly from M2 to M3 (TC: *F* = 17.871, *p* < 0.001 → *F* = 14.866, *p* < 0.001; HQ: *F* = 14.533, *p* < 0.001 → *F* = 12.593, *p* < 0.001; W and J: *F* = 1.860, *p* = 0.135 → *F* = 4.571, *p* < 0.001). Different physical activities and exercise forms can have an effect on subjective well-being, and the coefficients of different physical activity × exercise form have also reached significant levels (TC × EF: B = 0.006, *p* < 0.05; HQ × EF: B = 0.006, *p* < 0.05; W and J × EF: B = 0.009, *p* < 0.001), indicating that exercise form play a positive regulatory role when different physical activities affect subjective well-being; thus, H3 of this paper is supported.

**Table 7:** Testing the moderation roles of exercise form.

	Variable	Model 1		Model 2		Model 3	
		B	*t*	B	*t*	B	*t*
**TC**	Constant	5.284***	15.306	5.295***	15.330	5.253***	15.224
	TC	0.022***	7.174	0.022***	7.22	0.021***	6.577
	EF			−0.051	−1.025	−0.063	−1.271
	TC × EF					0.006*	2.362
	*R* ^2^	0.058		0.059		0.064	
	Adjusted *R*^2^	0.052		0.052		0.057	
	*F*-value	10.925***		9.285888		8.744***	
	∆*R*^2^	0.058		0.001		0.005	
	*∆F* value	10.925***		1.081, *p* = 0.299		5.228*	
**HQ**	Constant	5.204***	15.008	5.210***	15.014	5.172***	14.935
	HQ	0.02***	6.492	0.02***	6.491	0.018***	6.054
	EF			−0.031	−0.623	0.03	0.539
	HQ × EF					0.006*	2.551
	*R* ^2^	0.047		0.048		0.055	
	Adjusted *R*^2^	0.042		0.041		0.047	
	F-value	8.865***		7.450***		7.359***	
	∆*R*^2^	0.047		0.000		0.007	
	*∆F* value	8.865***		0.406, *p* = 0.524		6.534*	
**W and J**	Constant	5.190***	14.653	5.196***	14.659	5.220***	14.819
	W and J	0.006*	2.041	0.006	2.042	0.005	1.52
	EF			−0.031	−0.611	0.066	1.144
	W and J × EF					0.009***	3.554
	*R* ^2^	0.007		0.008		0.021	
	Adjusted *R*^2^	0.002		0.001		0.014	
	F-value	1.308, *p* = 0.258		1.152, *p* = 0.330		2.788**	
	∆*R*^2^	0.007		0.000		0.014	
	*∆F* value	1.308, *p* = 0.258		0.378, *p* = 0.539		12.512***	

B, unstandardized coefficient; dependent variable is subjective well-being; TC, tai chi; HQ, health qigong; W and J, walking and jogging; EF, exercise for.

* *p* < 0.05;

** *p* < 0.01;

*** *p* < 0.001.

**Figure 3:**
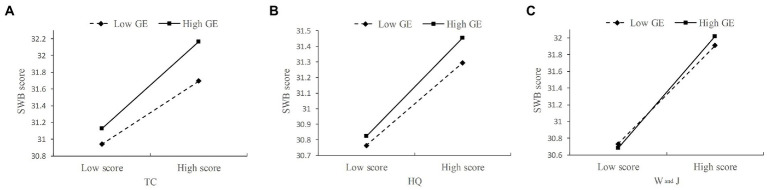
The moderation effect of exercise form between different physical activities and subjective well-being. **(A)** Simple slope analysis of the moderating effect of practice form on the relationship between tai chi and subjective well-being; **(B)** Simple slope analysis of the moderating effect of practice form on the relationship between health qigong and subjective well-being; **(C)** Simple slope analysis of the moderating effect of practice form on the relationship between walking and jogging and subjective well-being. SWB, subjective well-being; TC, tai chi; HQ, health qigong; W and J, walking and jogging; GE, group-based exercise.

In order to reveal the nature of the interaction effect and explain more clearly the regulatory role of exercise form in different physical activities and subjective well-being, according to the method suggested by Aiken et al. (1991), in this study a standard deviation (SD) is added to and subtracted by the means of exercise form, and groups with a high score or a low score were formed and performed a simple slope test. The results of the test (Figure 3) showed that different physical activities had a significant effect on subjective well-being, whether in the case of group or individual exercise. However, the slope of collective physical exercise is greater than that of individual exercise, which indicates that for older adults who prefer collective physical exercise (M + 1SD) different physical activities have a significant positive predictive effect on their subjective well-being; for older adults who do not have a strong sense of group-based exercise (M-1SD), although different physical activities also had a positive predictive effect on their subjective well-being, their predictive effect is minor, which indicates that with more engagement in group-based exercise, the predictive effect of different physical activities on the subjective well-being of older adults is gradually increased, proving that H3 is valid. In addition, as shown in Table 8, by comparing the slopes of different physical activities, it can be concluded that group walking and jogging has the strongest regulatory effect, followed by tai chi, and then health qigong.

**Table 8:** The slope of different physical activities.

	TC	HQ	W and J	*p*
High collectivity	0.010	0.006	0.013	***
Low collectivity	0.008	0.005	0.012	***

TC, tai chi; HQ, health qigong; *W and J*, walking and jogging.

*** *p* < 0.001.

## Discussion

### Different physical activity and subjective well-being in older adults

This paper mainly studied the relationship between the participation of different physical activities and subjective well-being among older adults in China, and the results showed that tai chi, health qigong, and walking and jogging all had a direct effect on the subjective well-being of older adults This is consistent with the findings of previous studies (Gillick, 1984; Li et al., 2001; Chow et al., 2012; Singleton, 2019). It is worth recalling that this study found the subjective well-being of older adults who practice tai chi to be the highest, maybe because during the normalization of pandemic prevention and control, a large number of basic studies found that clinical exercises based on aerobic breathing training can reduce airway resistance, increase lung function, improve body balance, improve immunity and quality of life (Mohamed and Alawna, 2020). Tai chi combines martial arts with health maintenance, and the adjustment of technical movements and mental breathing can effectively regulate and enhance the functional body systems of older adults. The gentle, smooth, slow, and natural technical characteristics of tai chi avoid damage caused by older adults’ participation in strenuous exercise, and the unpleasant emotions of life will also be released in the process of tai chi practice, and older adults’ overall evaluation of their own happiness level will be significantly improved. In addition, the study further concluded that physical activity is positively associated with various dimensions of subjective well-being in older adults. Studies have suggested that, in general, there is an inverse relationship between exercise intensity and positive affective responses; that is, the greater the intensity of exercise, the less pleasure is obtained (Ekkekakis et al., 2011). Intense physical activity may have greater health benefits but may also adversely affect mental health (Asztalos et al., 2009). Wicker and Frick (2015) analyzed survey data from 28 European countries and found that for people who participated in moderate-intensity exercise, the higher the frequency of exercise they did in the morning and the longer the time, the higher the level of subjective well-being. At the same time, for people who participated in high-intensity exercise or a mixture of moderate-intensity exercise and high-intensity exercise, exercise intensity and duration had a significant negative impact on subjective well-being. That is, high exercise intensity or excessive duration of exercise would reduce subjective well-being. Reed and Buck (2009) found that recreational physical activity programs with low-intensity (about 30% VO_2_max, 30–35 min/cycle, 3–5 days/week, and 10–12 weeks) had the strongest happiness effect in a comprehensive analysis of the effects of regular aerobic exercise on self-reported positive mood. Our study found that the subjective well-being and dimension scores of older adults who exercised moderately and with high intensity were higher than those in the low intensity group. This result further supports the idea that moderate- and high-intensity exercise has a more positive effect on the subjective well-being of older adults than lower-intensity exercise.

### The mediation effect of outdoor exercise environment

The research demonstrates that outdoor exercise environment, as an intermediary variable, plays an intermediary role between physical activity and subjective well-being. Wolf and Wohlfart (2014) believe that outdoor exercise environment can better provide physical activity opportunities for older adults and can also promote their mental health. The research found that the needs of different physical activities in the outdoor exercise environment played a positive guiding role in the subjective well-being of older adults. This is consistent with the results of previous studies (Sugiyama and Thompson, 2007; Loureiro and Veloso, 2014). In addition, the study also found that different physical activities have different needs for outdoor exercise environment, of which walking and jogging has the highest demand for outdoor exercise environment, followed by tai chi and health qigong. One possible explanation is that during the normalization of the pandemic, the places where older adults can exercise are restricted, and the demand for the exercise environment will change accordingly. Among all the sports, walking and jogging depends least on exercise conditions, and it is also the most common outdoor exercise (Stodolska et al., 2010). From the perspective of environmental psychology (Gehl, 2002), when people are in a good natural environment and living environment, they will spontaneously choose the appropriate outdoor exercise out of their own mentality or preference. In addition, for obese older adults, tai chi and health qigong cannot produce satisfactory effects enhancing cardiopulmonary function (Wang et al., 2004; Burini et al., 2006) due to insufficient exercise intensity, in contrast to walking and jogging, which can. In short, brisk walking and jogging, with relative exercise intensity, make higher demands on outdoor exercise environment. Lawton et al. (2017) argue that the exercise environment in which physical activity is performed is the most important factor for the production of mental health outcomes, and that people with exercise habits are more sensitive to the exercise environment, and the outdoor exercise environment is more conducive to reducing negative emotions and promoting mental health. Pretty et al. (2005) similarly mention that “green exercise” is more effective in improving cardiovascular and mental health than is exercise in unnatural environments. The results of the present paper further confirm this argument. Older adults who participate in physical activity generally have a higher frequency of exercise and belong to the “regular exercise population,” and the outdoor exercise environment plays a positive role in the subjective well-being of older adults.

### The regulation effect of exercise form

This study finds that exercise form play a positive regulatory role in the relationship between physical activity and subjective well-being; that is, when older adults participate in group-based exercise, the degree of influence of physical activity on subjective well-being is increased. This shows that when older people exercise in groups, physical activity has important implications for their subjective well-being. This is consistent with previous studies (Farrance et al., 2016), where the subjective well-being of older adults who participate in group activities is higher than that of older adults who participate in individual activities. Previous studies have also shown that regular, planned, and organized physical activity can effectively improve the emotional status of older adults (Komatsu et al., 2017). Possible explanations are as follows. First, organized exercise teams are usually equipped with exercise knowledge and can guide the members to choose exercises that match their cardiopulmonary function considering their own health status and underlying diseases, so that older adults can get a good exercise experience and wellbeing benefits. In addition, in the process of exercise, older adults have more opportunities for communication. They can search for common topics, cultivate common interests, which not only strengthens neighborhood relations within a community and increase the sense of social identity, but also plays an important role in their personal life satisfaction, mental health, and the resolving of setbacks (McAuley et al., 2000). In addition, the more older people engage in collective physical exercise, the more actively they face life and the lower their level of anxiety and depression are (Sebastião and Mirda, 2021). The intrinsic induction of mutual attraction and resonance that arises in interaction also helps to enliven the atmosphere and heighten the feeling of pleasure. Ku et al. (2016) and others believe that group walking can improve the subjective well-being of older adults, and older adults with higher activity frequency have higher levels of well-being. This result further supports the regulatory effect of collective exercise on the subjective well-being of older adults.

## Influences

This article makes some contributions to the mental health of older adults. First, the roles of walking and jogging, tai chi, and health qigong in enhancing subjective well-being are discussed by linking outdoor exercise environment, exercise form, and subjective well-being, in a more detailed way than in previous studies. By dividing physical activities into walking and jogging, tai chi, and health qigong, the relationship between different physical activities and subjective well-being is discussed more clearly. Secondly, the study used the AMOS path analysis method, evaluating the relationship between different physical activities and subjective well-being by using the outdoor exercise environment as the mediation variable within the mediation model, while the regulatory effect of exercise form (group and individual) was analyzed through linear regression. Finally, the study clarifies the relationship between different physical activities and subjective well-being and its internal mechanisms, and provides new theoretical insights and practical guidance for study of how to improve the subjective well-being of older adults. The authors argue that in order to improve the subjective happiness index, older adults should cultivate a sense of engaging in long-term physical activities, and that older adults’ moderate- and high-intensity exercise in outdoor environment is more conducive to improving the subjective well-being index, while encouraging older adults to participate in regular, organized, and planned group-based exercise.

## Limitations and future research directions

This study defines the relationship between different physical activities and subjective well-being and its internal mechanism, which has theoretical and practical significance. However, this study also has some limitations. First of all, this study is an investigation against the background of the long-term control and prevention of the COVID-19 pandemic. Large-scale public health events like this are not common, and under the influence of such events, the research results are also unusual. Second, physical activities include all types of activities, such as activities related to sports, leisure and occupation, general activities in daily life, and non-sedentary behaviors broadly (Caspersen et al., 1985). This paper only refers to the physical exercises that older adults participate in daily, ignoring other types of physical activities. Future research can focus on other types of physical activities of older adults, such as daily work, housework and gardening, transportation and leisure. In addition, there are certain limitations at the methodological level. This paper uses the PARS-3 scale to measure the physical exercise that older adults participate in daily from the three dimensions of exercise intensity, time and frequency, but fails to consider the measurement of other types of physical activities. In future research, the international physical activity questionnaire can be augmented to investigate different types of physical activities. At present, researchers have accurately pointed out that more accurate qualitative, intuitive, and quantitative methods need to be used to improve the measurement of subjective well-being (Nima et al., 2020). In this paper, the MUNSH scale is used to investigate the subjective well-being of older adults based on the four dimensions of PA, NA, PE and NE, there is a lack of providing direct measurement of the subjective well-being of older adults. In future research, we can consider adding the positive affect negative affect schedule, satisfaction with life scale, harmony in life scale, etc., so as to intuitively, accurately and comprehensively evaluate the subjective well-being of older adults. In-depth interviews with participants can also be implemented to supplement the gaps in qualitative research methods. Finally, this study adopts a cross-sectional model, which means that the research results cannot be compared at different time points, so the evidence is limited in this regard. In the future, research on different physical activities and subjective well-being of older adults can be tested through follow-up design and experimental research. At the same time, there are many internal mechanisms between different physical activities and subjective well-being in older adults. For example, sociological factors (including living areas, social support, interpersonal relationships, etc.) can be considered. The model needs to be further expanded in future research.

## Conclusion

The present study is unique in that it examines the relationship between different physical activities, outdoor exercise environment, exercise form, and subjective well-being in a sample of Chinese older adults. This study found that different physical activity types were positively correlated with the subjective well-being of older adults, such that older adults who practiced tai chi had the strongest subjective well-being. The outdoor exercise environment was identified as a mediator of different physical activities and subjective well-being, the exercise form can also moderate the impact of different physical activities on the subjective well-being of older adults. That is to say, the environment and the collective practice method will improve the subjective well-being of older adults. The identification of the correlation path between different physical activities and subjective well-being provides evidence to clarify the relationship between different physical activities and the subjective well-being of older adults. This study provides a reference for mental health intervention in older adults.

## Data availability statement

The original contributions presented in the study are included in the article/supplementary material, further inquiries can be directed to the corresponding author.

## Author contributions

XM and BZ: conceptualization and project administration. XM and FZ: methodology. XM and QY: formal analysis, investigation, and writing—original draft preparation. XM, FZ, and QY: validation. XM: data curation and funding acquisition. XM, GJ, and YT: writing—review and editing. All authors contributed to the article and approved the submitted version.

## Funding

This research was funded by Sichuan Federation of Social Sciences Associations, grunt number SC21ZW003.

## Conflict of interest

The authors declare that the research was conducted in the absence of any commercial or financial relationships that could be construed as a potential conflict of interest.

## Publisher’s note

All claims expressed in this article are solely those of the authors and do not necessarily represent those of their affiliated organizations, or those of the publisher, the editors and the reviewers. Any product that may be evaluated in this article, or claim that may be made by its manufacturer, is not guaranteed or endorsed by the publisher.
